# Mechanism of P-Hydroxy Benzyl Alcohol Against Cerebral Ischemia Based on Metabonomics Analysis

**DOI:** 10.3390/ijms26010317

**Published:** 2025-01-01

**Authors:** Tian Xiao, Xingling Yu, Jie Tao, Jiaoyang Tan, Zhourong Zhao, Chao Zhang, Xiaohua Duan

**Affiliations:** Yunnan Key Laboratory of Dai and Yi Medicines, Yunnan University of Chinese Medicine, Kunming 650500, China; xiaot7107@163.com (T.X.); 18212943765@163.com (X.Y.); 19188572767@163.com (J.T.); 13350039866@163.com (J.T.); zhaozr_cpu@163.com (Z.Z.); zhangc19@21cn.com (C.Z.)

**Keywords:** p-hydroxybenzyl alcohol, metabonomics, mitochondrial dysfunction, cognitive dysfunction, cerebral ischemia

## Abstract

Stroke is the leading cause of death and disability worldwide, with ischemic stroke accounting for the majority of these. HBA is the active ingredient in *Gastrodia elata* and has potential therapeutic effects on central nervous system diseases. In this study, the cell model of cerebral ischemia was replicated by the culture method of oxygen-glucose deprivation/reoxygenation, and the rat model of vascular dementia was established by the two-vessel occlusion method. Metabolomics technology was employed to analyze the metabolic changes in ischemic neurons induced by HBA, and potential therapeutic targets were verified. The protective effects of HBA on ischemic neurons and their mitochondria were examined through multiple indicators, and the related mechanisms were verified. HBA can improve post-ischemic cognitive impairment in rats, and its mechanism is related to the regulation of the choline-activated phospholipase D2/Sirtuin 1/peroxisome proliferator-activated receptor-γ coactivator 1α pathway to improve mitochondrial function and reduce autophagic activity to maintain mitochondrial homeostasis. It is concluded that HBA has a protective effect on neuronal damage and cognitive impairment caused by cerebral ischemia by regulating key metabolites and signaling pathways, and that it provides a new molecular target for the treatment of cerebral ischemia.

## 1. Introduction

According to the 2019 Global Burden of Disease Risk Factors study analysis, stroke remains the second leading cause of death globally (11.6% of total deaths) and the third leading cause of disability (5.7% of actual disabilities), of which ischemic stroke accounts for 62.4% of cases [1]. Intravenous thrombolysis and mechanical thrombectomy are commonly used in the clinical treatment of ischemic stroke [2]. These treatments have limitations such as a narrow time window and the inability of patients to meet the indications for surgery [3]. At the same time, after recanalization treatment, ~1/3 of patients have an increased infarct size, no reflow or early deterioration of neurological function. This condition is mainly due to the aggravation of pathological phenotypes, such as mitochondrial dysfunction, oxidative stress and apoptosis in the process of restoring blood flow [4,5]. Stroke also increases the risk of developing vascular dementia, with about one-third of stroke patients eventually developing dementia, most of whom develop vascular dementia (VD) [6,7,8]. The hippocampus, a deep structure of the brain closely related to learning, memory, and cognitive function, is highly sensitive to hypoxia/ischemic injury. Vascular damage to the hippocampus is thought to be a key factor in memory dysfunction during healthy aging and cerebrovascular disease. Alleviating hippocampal damage is essential for the development of effective treatments to slow cognitive decline [9].

Therefore, developing drugs to reduce pathological damage after thrombolysis is essential to improve the treatment of ischemic stroke. Because it is a multi-component important condition, traditional Chinese medicine is expected to be effective in the treatment of diseases with multiple pathological mechanisms such as antioxidative stress [10].

p-hydroxybenzyl alcohol (HBA) is not only one of the main active components of *Gastrodia elata*, a valuable Chinese herbal medicine, but it is also listed as a quality detection index component of *Gastrodia elata* in the 2020 edition of the Chinese Pharmacopoeia [11]. Studies have indicated that HBA has therapeutic potential in the central nervous system. For instance, HBA may prevent cognitive impairment in the Aβ42 oligomer-induced Alzheimer’s disease mouse model by increasing neurotrophic and reducing inflammatory factors [12], and it also helps to reduce scopolamine-induced learning and memory impairment and increase endogenous neuronal proliferation in the brain [13]. Studies on the effect of HBA on ischemic stroke have found that the administration of HBA 30 min prior to focal ischemia significantly reduced the volume of cerebral infarction, which was indicated to be related to lowering apoptosis and enhancing antioxidation [14,15,16]. HBA may also improve brain injury in rats with middle cerebral artery occlusion (MCAO) by enhancing the protective effect against Zn^2+^ toxicity in neurons and astrocytes [17]. The aforementioned studies fully reflect the broad therapeutic potential of HBA in treating ischemic stroke.

Metabonomics was developed at the end of the last century to study the number of and changes in endogenous metabolites in an organism [18]. Currently, metabonomics is being widely applied in drug target screening, disease diagnosis, and personalized drug therapy. It is mainly used to analyze differential metabolites between experimental and control groups to reveal the physiological and pathological mechanisms [19]. Numerous studies have applied metabonomics analyses to the study of ischemic stroke. Ma et al. [20] found that Edaravone (EDA) injection significantly increased the level of taurine in a mouse model of MCAO/reperfusion (MCAO/R). Further analysis confirmed that the rate-limiting enzyme in taurine production, cysteine sulfonic acid decarboxylase, significantly increased after EDA treatment and inhibited endothelial cell apoptosis. Zhou et al. [21] found that Danshen Chuanxiongqin injection, a traditional Chinese medicine compound injection, attenuated neuronal inflammation and apoptosis in a rat model of MCAO/R by inhibiting the activation of the sphingosine kinase 1/sphingosine-1-phosphate axis. In addition, Luo et al. [22] found that 3,4-dihydroxybenzaldehyde improved mitochondrial dysfunction by activating O-linked β-N-acetylglucosamine transferase and inhibiting neuronal apoptosis induced by cerebral ischemia–reperfusion injury. Therefore, analyzing differential metabolites using metabonomics may clarify the mechanism of drug action and provide a rapid and direct method for discovering pharmacological means, as well as a basis for clinical treatment. However, previous studies did not explore the potential value of HBA for ischemic stroke in terms of its effects on metabolism, to the best of our knowledge.

Therefore, in the present study, the ultra-high-performance liquid chromatography coupled with mass spectrometry (UPLC/MS) metabonomics method was employed to characterize the effects of HBA on the metabolic characteristics of oxygen-glucose deprivation/reoxygenation (OGD/R) in the mouse hippocampal neuronal cell line HT22. Finally, target metabolic analysis was performed, and potential therapeutic targets were verified by Western blotting, reverse transcription-quantitative PCR (RT-qPCR), and electron microscopy. The underlying mechanism of action was tested by replicating vascular dementia (VD) rats with two-vessel occlusion (2VO). This study will reveal the underlying mechanism of HBA in the treatment of cerebral ischemia through metabolomic analysis, especially the role of improving mitochondrial function and regulating metabolic pathways, which provides a new strategy and theoretical basis for the treatment of cerebral ischemia.

## 2. Results

### 2.1. Protective Effect of HBA in OGD/R Cells

**Figure 1:**
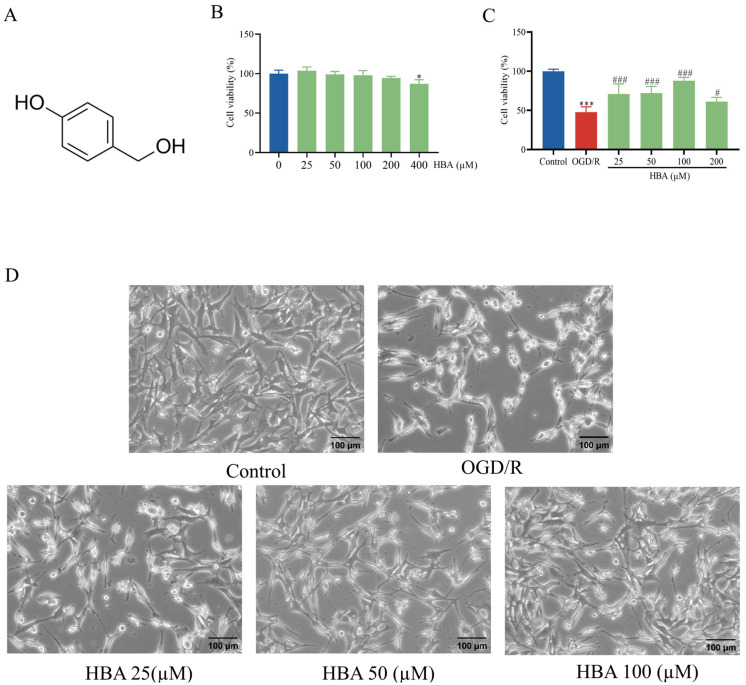
Protective effect of HBA on OGD/R damage to HT22 cells. (**A**) Chemical structure of HBA. (**B**) Effect of HBA on the viability of HT22 cells (n = 6). (**C**) Protective effect of HBA on HT22 cells induced by OGD/R (n = 6). (**D**) Representative image indicating morphological changes induced by OGD/R and the protective effect of HBA; the model group featured a large number of cells with shrinkage, a reduction in cell processes and cytoplasmic condensation, while the morphology of cells in the HBA group was improved (magnification, ×400). Values are expressed as the mean ± standard error of the mean. *** *p* < 0.001, * *p* < 0.05 vs. Control group; ### *p* < 0.001, # *p* < 0.05 vs. OGD/R group. HBA, p-hydroxybenzyl alcohol; OGD/R, oxygen-glucose deprivation/reoxygenation.

HBA is a phenolic compound with a chemical structure comprising a benzene ring and two hydroxyl groups (Figure 1A). First, the effect of HBA on the viability of HT22 cells was evaluated. The results indicate that exposure to HBA at concentrations of 12.5–200 μM did not significantly impact the survival rate of HT22 cells when compared to the 0 μM group (Figure 1B). Next, the protective effect of HBA (in amounts of 25, 50, 100, or 200 μM) against OGD/R damage was evaluated. After OGD/R treatment, compared with the OGD/R group, the survival rate of HT22 cells decreased significantly (*p* < 0.001). Compared with the OGD/R group, the HBA groups had an increased cell survival rate, respectively (*p* < 0.001, *p* < 0.05, Figure 1C). In addition, cell morphological observation indicated that in the OGD/R group, a large number of cells had shrunk. Furthermore, compared with the control group, the OGD/R group exhibited decreased cellular processes and condensed cytoplasm. Of note, in the HBA groups, improvements in the morphology of cells were observed compared with the model group (Figure 1D). These results suggested that HBA was able to ameliorate OGD/R injury to cells. As the cell survival rate was the highest and OGD/R damage was effectively reduced at 100 μM HBA, this concentration was selected for the follow-up metabonomics analysis.

### 2.2. Analysis of Metabolites in OGD/R Cells After HBA Treatment

**Figure 2:**
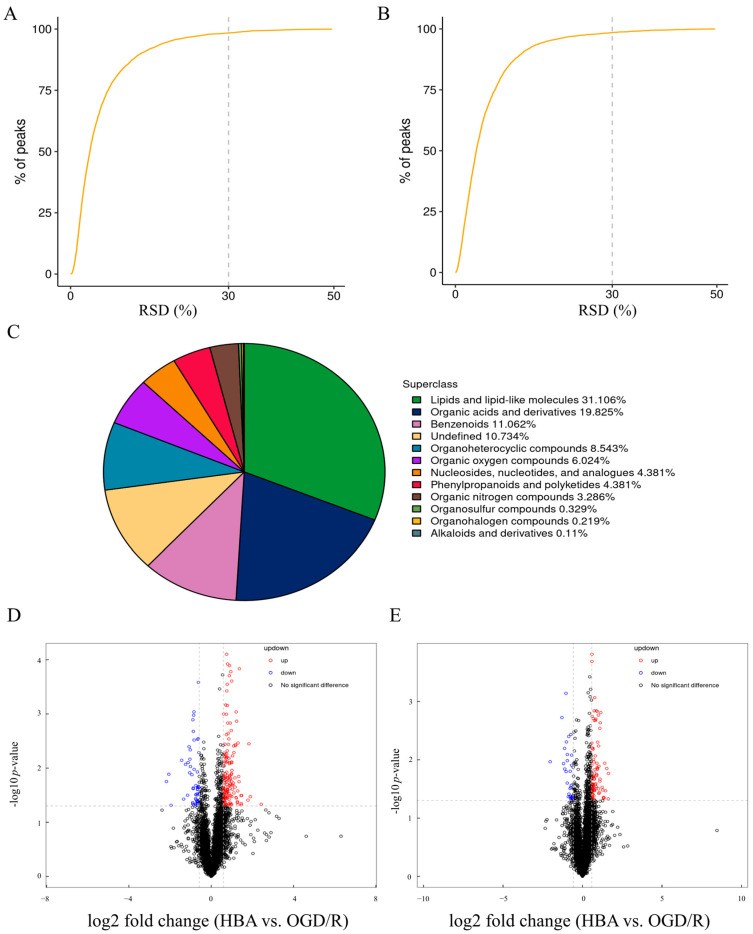
Metabolic analysis comparison between the OGD/R group and HBA group. (**A**) RSD of the QC samples in positive mode. (**B**) RSD of the QC samples in negative mode. (**C**) Pie chart presenting the proportion of identified metabolites in each chemical classification. (**D**) Volcano plots of differential metabolites of positive mode. (**E**) Volcano plots of differential metabolites of negative mode. The significantly upregulated metabolites are presented in red, the significantly downregulated metabolites are presented in blue, and the insignificantly changed metabolites are presented in black. HBA, p-hydroxybenzyl alcohol; OGD/R, oxygen-glucose deprivation/reoxygenation; RSD, relative standard deviation.

The better the instrument stability, indicated by a smaller relative standard deviation (RSD) of ion peak abundance in QC samples, the higher the data quality [23]. RSD results of QC samples indicated that the number of peak intensities with RSD ≤ 30% among the QC samples accounted for >80% of the total number of peak intensities of all QC samples (Figure 2A,B). This indicated that the instrument’s analysis system had good stability and that the data may be used for further analysis. A total of 913 metabolites were identified through the combination of positive and negative ion patterns, mainly belonging to chemicals, such as lipids and lipid-like molecules (31.106%), organic acids and derivatives (19.825%), benzenoids (11.062%), undefined (10.734%), and other categories (Figure 2C). Differences between the OGD/R group and the HBA groups were analyzed. The differences between all metabolites (including unidentified metabolites) with FC > 1.5 or FC < 0.67 and *p* < 0.05 were visualized in the form of a volcano plot (Figure 2D,E), which indicated that there were significant inter-group differences.

### 2.3. Enrichment Analysis of Differential Metabolites and Their Metabolic Pathways in OGD/R Cells

**Figure 3:**
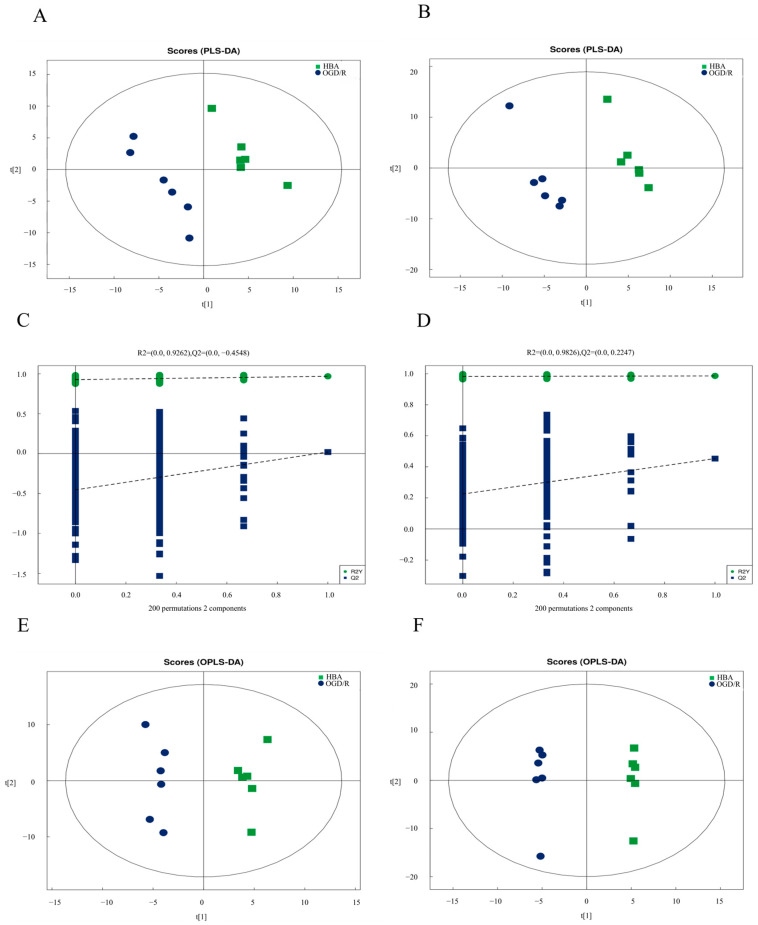
Differences in metabolites between the OGD/R group and the HBA group. (**A**) positive and (**B**) negative ion mode PLS-DA score chart, in which abscissa t[1] represents principal component 1, ordinate t[2] represents main component 2, and the ellipse represents the 95% confidence interval. Symbols of the same color represent the biological repetition within the group and the distribution of the symbols reflects the degree of differences between and within groups. (**C**) Positive and (**D**) negative ion mode PLS-DA permutation test chart, in which the abscissa represents the degree of replacement retention, namely the proportion consistent with the order of Y variables in the original model, and the ordinate represents the values of R2 and Q2. The green dots indicate R2, the blue squares indicate Q2, and the two dotted lines represent the regression lines of R2 and Q2. The R2 and Q2 in the upper right corner suggest that the replacement retention equals 1, namely the R2 and Q2 values of the original model. (**E**) Positive and (**F**) negative ion mode OPLS-DA score diagram. In the figure, the abscissa t[1] represents principal component 1, the ordinate t[1] represents main component 2, and the ellipse represents the 95% confidence interval. Symbols of the same color represent the biological repetition within the group and the distribution of the symbols reflects the degree of differences between and within groups. HBA, p-hydroxybenzyl alcohol; OGD/R, oxygen-glucose deprivation/reoxygenation; PLS-DA, partial least squares discrimination analysis; OPLS-DA, orthogonal PLS-DA.

First, the relationship between metabolite expression and sample category was analyzed using PLS-DA. The results indicated that the PLS-DA model could be used to distinguish between the two groups of samples (Figure 3A,B). To avoid the over-fitting of the PLS-DA model in the modeling process, the replacement test was used to ensure the validity of the model. The results indicated that there was no over-fitting in the original model and that the model was robust (Figure 3C,D). To screen for differential metabolites, OPLS-DA was used (Figure 3E,F) to obtain variable VIP values, which were used to measure the influence and explanatory ability of the metabolite expression patterns on the classification and discrimination of each group, and to mine biologically significant differential metabolites. In the present study, VIP > 1 and *p* < 0.05 were used as the screening criteria to indicate substantial differences in metabolites. There were 17 significantly different metabolites between the HBA group and the OGD/R group. Of these metabolites, 15 were upregulated. The other two metabolites were downregulated (Table 1).

**Figure 4:**
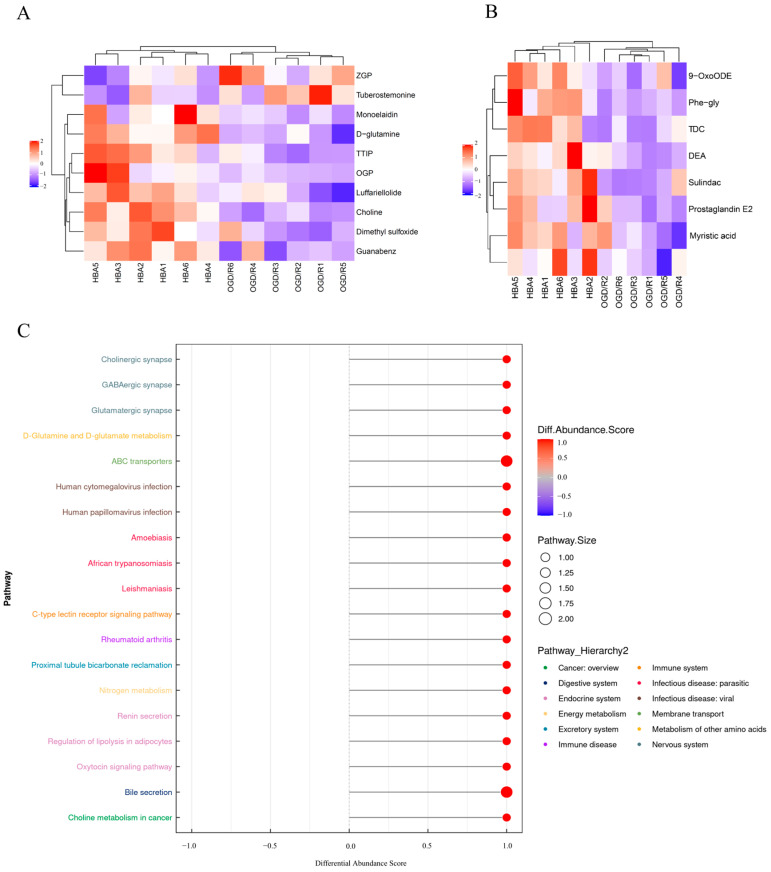
Enrichment analysis of metabolic pathways of differential metabolites. Hierarchical clustering heat map of metabolites indicating a significant difference between (**A**) positive and (**B**) negative ion patterns. Each row represents a differential metabolite and each column represents a group of samples. Red represents relatively high expressions; blue represents relatively low expression, and metabolites with similar expression patterns are clustered under the same cluster on the left. (**C**) DA score map of all metabolic enriched pathways, in which the Y axis represents the name of the differential pathway and the X axis represents the DA score. A DA score of 1 indicates an upward trend and that of −1 indicates a downward trend in the expression of all identified metabolites in the pathway. The length of the segment represents the absolute value of the DA score and the size of the circle at the end of the line indicates the number of metabolites in the pathway. ZGP, 1-(1z-hexadecenyl)-sn-glycero-3-phosphocholine; TTIP, 3,3,4-trimethy-l-[24]-4-penten-1-one-1; OGP, oleoyl-sn-glycero-3-phosphocholine; TDC, tetrahydrocorticosterone; DEA, 11,12-dihydroxy-5z,8z,14z-eicosatrienoic acid; HBA, p-hydroxybenzyl alcohol; OGD/R, oxygen-glucose deprivation/reoxygenation; DA, differential abundance.

To examine the association between the different samples and the expression patterns of metabolites more comprehensively and intuitively, a cluster analysis was performed on the selected metabolites. Metabolites in the same cluster had similar expression patterns and may have similar functions or participate in the same metabolic process or cellular pathway (Figure 4A,B). To observe the changes in metabolism overall, the average differences in all metabolites were examined using the differential abundance score in each KEGG pathway. This demonstrates that the differential metabolites between the HBA and OGD/R groups interact mainly through ‘cholinergic synapse’, ‘GABAergic synapse’, and ‘glutamatergic synapse’, as well as different pathways, which jointly affect the ‘nervous system’, ‘excretory system’, ‘energy metabolism’, and other systems of the body (Figure 4C).

### 2.4. Verification of PLD2 Expression After HBA Treatment in OGD/R Cells

**Figure 5:**
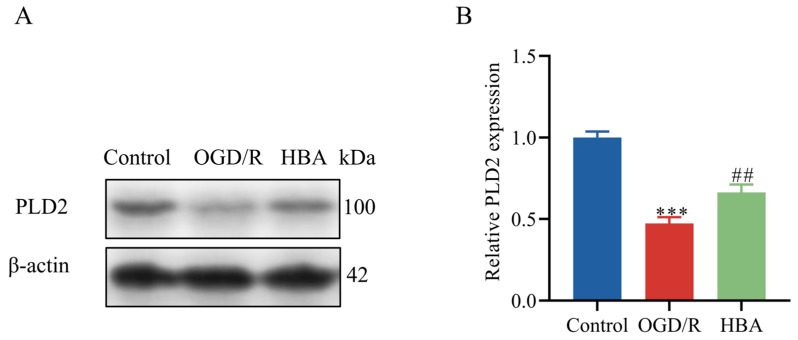
Effect of HBA on PLD2 in OGD/R cells. (**A**) Representative Western blot and (**B**) semi-quantitative analysis of PLD2 expression. Values are expressed as the mean ± standard error of the mean (n = 3). *** *p* < 0.001 vs. Control group; ## *p* < 0.01 vs. OGD/R group. PLD2, phospholipase D; HBA, p-hydroxybenzyl alcohol; OGD/R, oxygen-glucose deprivation/reoxygenation.

Western blot analysis of PLD2 protein expression indicated a significant increase in the HBA group (*p* < 0.001) compared with that in the OGD/R group (*p* < 0.001) (Figure 5A,B). This suggested that in cells treated with OGD/R and HBA, HBA increases the level of choline by promoting the expression of PLD2.

### 2.5. Inhibition of Mitochondrial Dysfunction After HBA Treatment in OGD/R Cells

**Figure 6:**
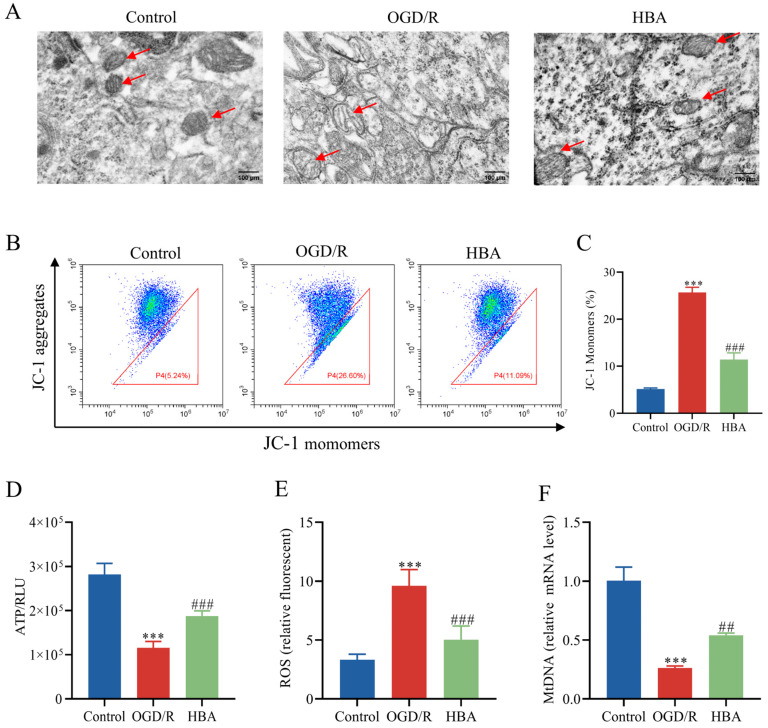
Effect of HBA on mitochondrial dysfunction induced by OGD/R. (**A**) Representative transmission electron microscopy images of mitochondria of HT22 cells. In the OGD/R group, the mitochondria (red arrows) were swollen, the cristae disappeared, and the membrane was damaged. By contrast, in the HBA group, the mitochondrial structure was intact and the cristae were transparent (magnification, ×20,000). Flow cytometry was used to detect the MMP. Green fluorescence represents the intensity of JC-1 monomer, and blue fluorescence represents the intensity of DAPI fluorescence. (**B**,**C**) Representative flow cytometry dot plot and quantitative results (n = 3). (**D**) ATP detection (n = 6). (**E**) ROS detection (n = 6). (**F**) mtDNA detection (n = 3). Values are expressed as the mean ± standard error of the mean. *** *p* < 0.001 vs. Control group; ### *p* < 0.001, ## *p* < 0.01 vs. OGD/R group. OGD/R, oxygen-glucose deprivation/reoxygenation; HBA, p-hydroxybenzyl alcohol; MMP, mitochondrial membrane potential; RLU, relative light unit; ROS, reactive oxygen species; mtDNA, mitochondrial DNA.

**Figure 7:**
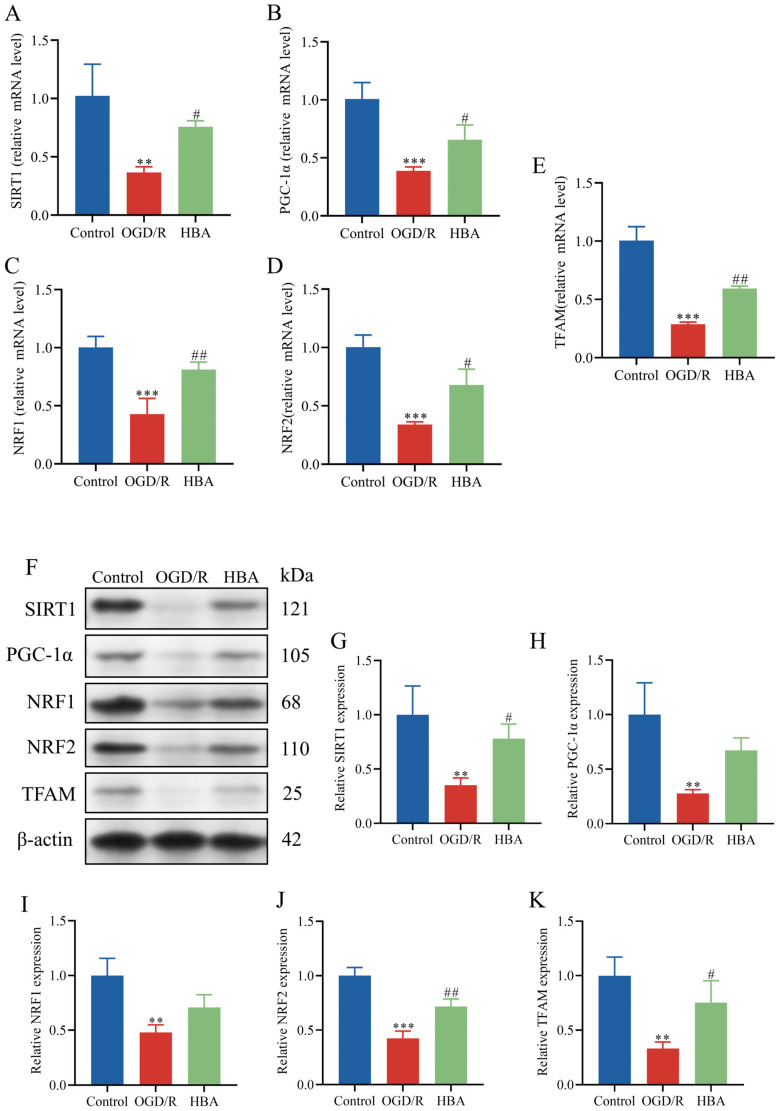
HBA ameliorates mitochondrial dysfunction through the SIRT1/PGC-1α signaling pathway. mRNA levels of (**A**) SIRT1, (**B**) PGC-1α, (**C**) NRF1, (**D**) NRF2, and (**E**) TFAM. (**F**) Representative Western blot and semi-quantitative analysis of the expression of (**G**) SIRT1, (**H**) PGC-1α, (**I**) NRF1, (**J**) NRF2, and (**K**) TFAM. Values are expressed as the mean ± standard error of the mean (n = 3). *** *p* < 0.001 and ** *p* < 0.01 vs. Control group; ## *p* < 0.01, # *p* < 0.05 vs. OGD/R group. OGD/R, oxygen-glucose deprivation/reoxygenation; HBA, p-hydroxybenzyl alcohol; SIRT1, sirtuin1; PGC-1α, peroxisome proliferator-activated receptor γ coactivator 1α; NRF, nuclear respiratory factor; TFAM, transcription factor A, mitochondrial.

Mitochondrial dysfunction may cause pathological changes, such as energy deficiency, oxidative stress, and apoptosis, and may aggravate brain injury after cerebral ischemia [4]. Using TEM, the swelling of mitochondria, damage to the mitochondrial membrane structure, and the disappearance of cristae were observed after OGD/R. However, after HBA treatment, the structure of the mitochondria was intact, and the cristae were evident (Figure 6A). Mitochondrial function indicators were then compared among the groups. Compared with the control group, after OGD/R, the level of ROS increased, and the content of ATP, mtDNA, and MMP decreased (*p* < 0.001). These deleterious changes were improved by HBA treatment: The level of MMP, ATP, and mtDNA increased (*p* < 0.001, *p* < 0.01), and the number of ROS decreased (*p* < 0.001) (Figure 6B–F). These results indicated that HBA had a protective effect on mitochondria. Next, the molecular mechanisms of mitochondrial function protection were studied. Compared with the control group, the RT-qPCR results indicated that OGD/R treatment decreased the relative mRNA levels of SIRT1, PGC-1α, NRF1, NRF2, and TFAM (*p* < 0.001, *p* < 0.01). Compared with the OGD/R group, after HBA, the levels of SIRT1, PGC-1α, NRF1, NRF2, and TFAM increased (*p* < 0.01, *p* < 0.05, Figure 7A–E). Similarly, compared with the control group, Western blot analysis suggested that in the OGD/R group, the relative protein expression levels of SIRT1, PGC-1α, NRF1, NRF2, and TFAM decreased (*p* < 0.001, *p* < 0.01). Compared with the OGD/R group, after treatment with HBA, the levels of SIRT1, PGC-1α, NRF1, NRF2, and TFAM increased (*p* < 0.01, *p* < 0.05, Figure 7F–K). Taken together, these results suggested that HBA may improve mitochondrial dysfunction and protect against ischemic injury through the SIRT1/PGC-1α signaling pathway.

### 2.6. Effect of HBA on Cognitive Memory Function in 2VO Rats

**Figure 8:**
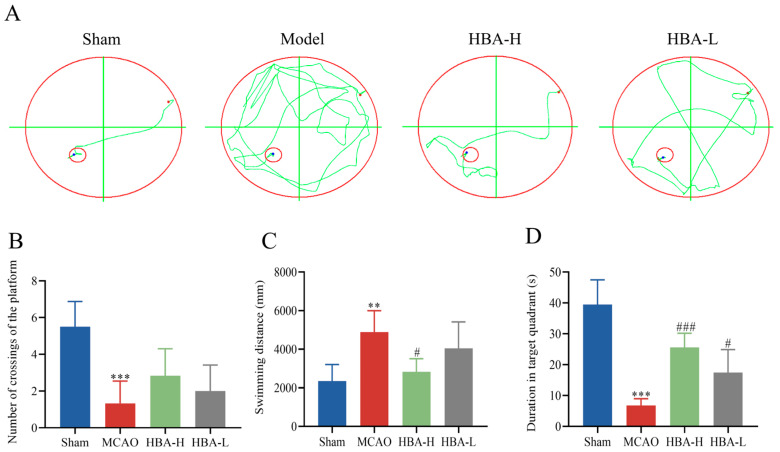
Effect of HBA on cognitive memory function in 2VO rats. (**A**) Swimming path in the Morris water maze experiment (n = 6). The dots represent the start of swimming and the circle represents the end of swimming. (**B**) Number of crossings of the platform in the Morris water maze experiment (n = 6). (**C**) Swimming distance in the Morris water maze experiment (n = 6). (**D**) Duration in the target quadrant in the Morris water maze experiment (n = 6). Values are expressed as the mean ± standard error of the mean. *** *p* < 0.001, ** *p* < 0.01 vs. Sham group; ### *p* < 0.001, # *p* < 0.05 vs. Model group. HBA, p-hydroxybenzyl alcohol.

In the Morris water maze experiment, after modeling, rats showed a significant decline in cognitive and memory function. In the positioning navigation experiment, their escape latency was significantly longer than that of the sham group, and the swimming path was also more tortuous and disordered (*p* < 0.001, *p* < 0.01). Situations such as wandering and entering the wrong quadrant often occurred. In the spatial exploration experiment, the number of platform crossings by rats in the 2VO group was significantly less than that in the sham group, and the proportion of time spent in the target quadrant was also greatly reduced (*p* < 0.001). After HBA treatment, the HBA-H group (20 mg/kg) and HBA-L group (10 mg/kg) were administered by gavage for 30 days, once a day. In the positioning navigation experiment, the escape latency of rats gradually shortened and approached the level of the sham group, and the swimming path also became relatively regular and efficient (*p* < 0.05). In the spatial exploration experiment, the number of platform crossings by rats in the HBA-H and HBA-L groups increased significantly, and the proportion of time spent in the target quadrant also increased significantly (*p* < 0.001, *p* < 0.05). Overall, this indicates that the cognitive and memory function of VD rats was effectively restored after HBA administration (Figure 8).

### 2.7. Effect of HBA on HE Staining Changes in the Hippocampus of 2VO Rats

**Figure 9:**
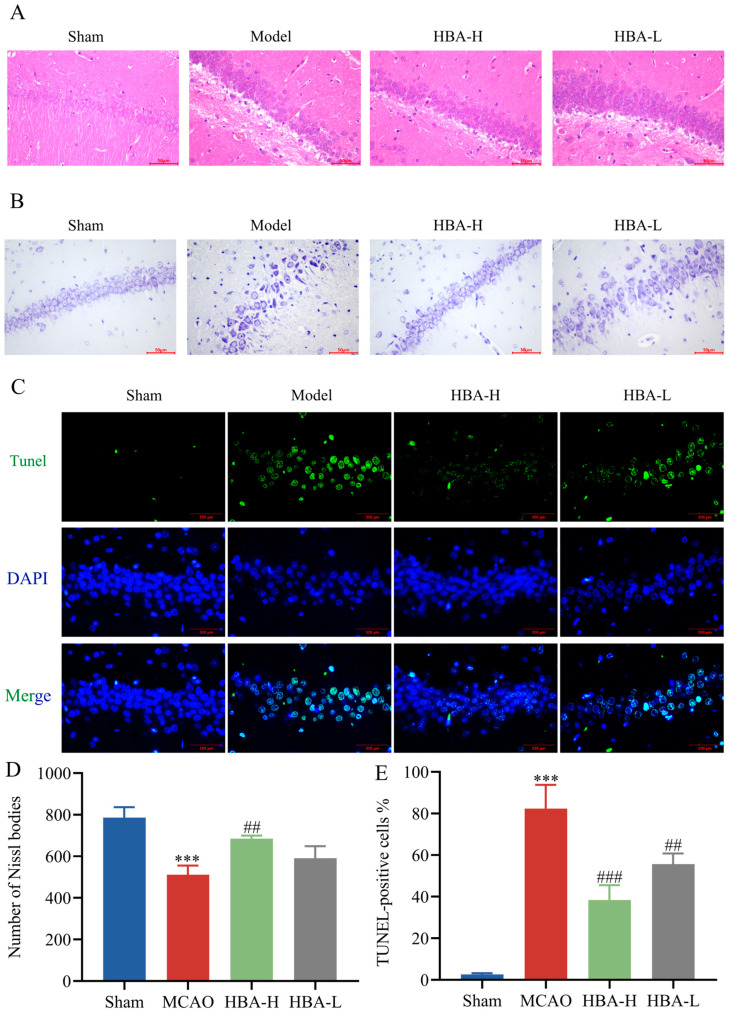
Effect of HBA on changes in the hippocampal tissue of 2VO rats (**A**) HE staining figures of HBA treating 2VO rats (n = 3). (**B**) Nissl staining figures of HBA treating 2VO rats. (**C**) TUNEL staining figures of HBA treating 2VO rats. (**D**) Changes in the number of Nissl bodies in 2VO rats treated with HBA (n = 3). (**E**) Changes in the proportion of TUNEL-positive cells in 2VO rats treated with HBA (n = 3). Values are expressed as the mean ± standard error of the mean. *** *p* < 0.001 vs. Sham group; ### *p* < 0.001, ## *p* < 0.01 vs. Model group. HBA, p-hydroxybenzyl alcohol.

After successful modeling, obvious morphological changes occurred in the hippocampal neurons of 2VO rats. Neurons changed from being closely and neatly arranged under normal circumstances to being sparse and disordered, with increased cell spacing. Characteristics of cell damage such as shrinkage, cell body reduction, and pyknosis and deep staining of the nucleus appeared. At the same time, the neuropil structure in the tissue became blurred, indicating that the integrity of nerve fibers was damaged. In addition, there was also the phenomenon of inflammatory cell infiltration in the hippocampal region of 2VO rats, suggesting the presence of an inflammatory reaction. After the administration of HBA, the above pathological state was effectively restored, and the morphology of the hippocampal region of rats tended to be normal (Figure 9A).

### 2.8. Effect of HBA on Changes in Nissl Staining in Hippocampal Tissue of 2VO Rats

After successful modeling, a reduction in the number of Nissl bodies in 2VO rats can be observed (*p* < 0.001). At the same time, abnormal morphologies such as cell body shrinkage and lighter staining appear in neurons. In addition, due to the disorder of the neuropil structure, the uniformity of Nissl staining is also disrupted. After administration, the number of Nissl bodies significantly increases (*p* < 0.001), the morphology of neurons is improved, the phenomenon of cell body shrinkage is alleviated, the staining becomes darker, and relatively normal morphological characteristics are restored (*p* < 0.01). The neuropil structure gradually becomes clear and orderly, and the uniformity of Nissl staining is also enhanced (Figure 9B,D).

### 2.9. Effect of HBA on Changes in TUNEL Staining in Hippocampal Tissue of 2VO Rats

After successful modeling, the proportion of TUNEL-positive cells in rats of the 2VO group was significantly increased (*p* < 0.001), which indicates that there is a large number of apoptotic phenomena. After the administration of HBA, the number of TUNEL-positive cells is effectively reduced (*p* < 0.001, *p* < 0.01), suggesting that the apoptotic phenomenon is inhibited and the survival of neurons is promoted (Figure 9C,E).

### 2.10. Effect of HBA on PLD2/SIRT1/PGC-1α Survival Signaling Axis in 2VO Rats

**Figure 10:**
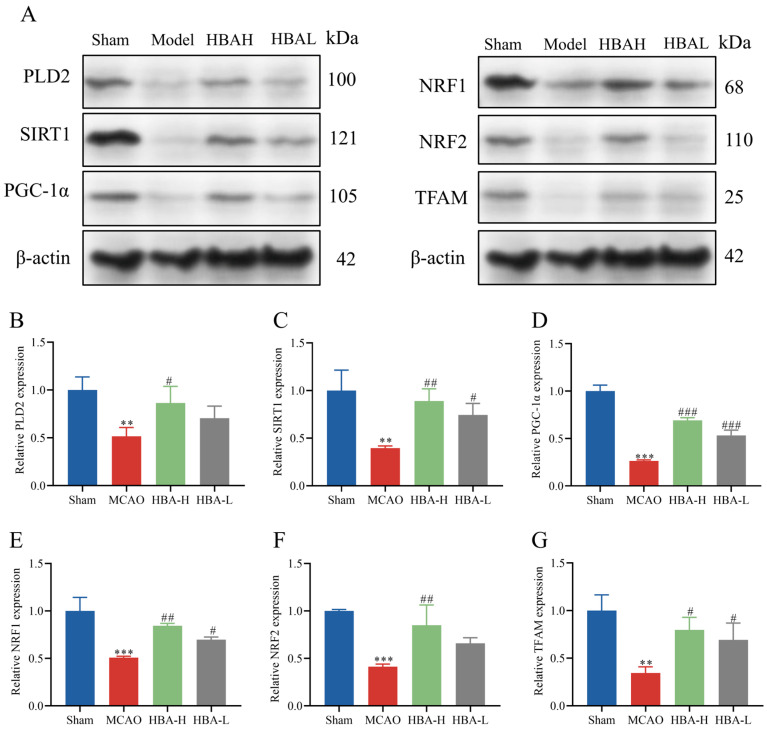
Effect of HBA on PLD2/SIRT1/PGC-1α survival signaling axis in 2VO rats. (**A**) Representative Western blot and semi-quantitative analysis of the expression of (**B**) PLD2, (**C**) SIRT1, (**D**) PGC-1α, (**E**) NRF1, (**F**) NRF2, and (**G**) TFAM. Values are expressed as the mean ± standard error of the mean (n = 3). *** *p* < 0.001, ** *p* < 0.01 vs. Sham group; ### *p* < 0.001, ## *p* < 0.01, # *p* < 0.05 vs. Model group. HBA, p-hydroxybenzyl alcohol.

Similarly, compared with the sham group, Western blot analysis suggested that in the model group, the relative protein expression levels of PLD2, SIRT1, PGC-1α, NRF1, NRF2, and TFAM were decreased (*p* < 0.001, *p* < 0.01). Compared with the model group, after treatment with HBA, the levels off PLD2, SIRT1, PGC-1α, NRF1, NRF2, and TFAM increased (*p* < 0.01, *p* < 0.05, Figure 10). The results suggested that HBA may improve mitochondrial dysfunction and protect against ischemic injury through the PLD2/ SIRT1/PGC-1α signaling pathway.

### 2.11. Effect of HBA on Mitophagic Activity in 2VO Rats

**Figure 11:**
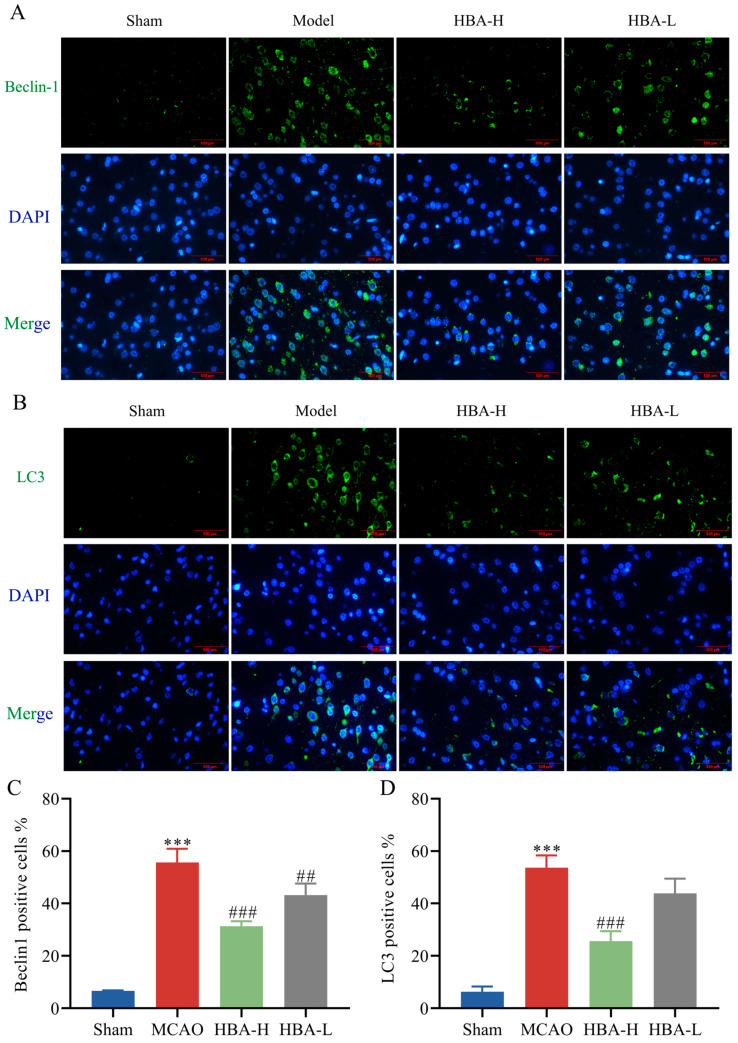
Effect of HBA on mitophagic activity in 2VO rats. Immunofluorescence staining figures of (**A**) Beclin1 and (**B**) LC3. The expression of (**C**) Beclin1 and (**D**) LC3. Values are expressed as the mean ± standard error of the mean (n = 3). *** *p* < 0.001 vs. Sham group; ### *p* < 0.001, ## *p* < 0.01 Model group. HBA, p-hydroxybenzyl alcohol.

**Figure 12:**
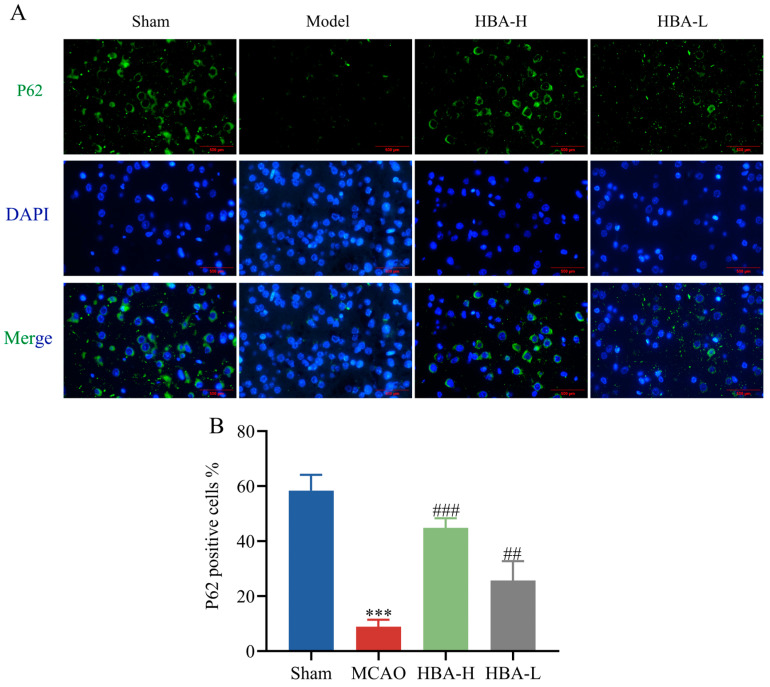
Effect of HBA on mitophagic activity in 2VO rats. (**A**) Immunofluorescence staining figures of P62. (**B**) The expression of P62. Values are expressed as the mean ± standard error of the mean (n = 3). *** *p* < 0.001 vs. Sham group; ### *p* < 0.001, ## *p* < 0.01 vs. Model group. HBA, p-hydroxybenzyl alcohol.

Compared with the sham group, immunofluorescence staining analysis suggested that in the model group, the relative protein expression levels of Beclin1 and LC3 were increased, and P62 was decreased (*p* < 0.001). Compared with the model group, after treatment with HBA, the levels of Beclin1 and LC3 were decreased, and P62 was increased (*p* < 0.001, *p* < 0.01) (Figure 11 and Figure 12).

## 3. Discussion

In the present study, the HT22 OGD/R model and metabonomics analysis and verification methods were used to explore the mechanism of HBA in protecting against ischemia–reperfusion injury in vitro. The results suggested that HBA may ameliorate the OGD/R injury of HT22 cells via the PLD2/SIRT1 pathway in vitro. Initially, it was confirmed that HBA at concentrations ranging from 12.5 to 100 μM was not cytotoxic and significantly increased the cell survival rate after OGD/R, effectively reducing the morphological damage to cells. Therefore, it was suggested that HBA has a protective effect on the OGD/R HT22 cell model. This provided a reliable basis for subsequent metabonomics research.

In the present study, the metabonomics analysis suggested that HBA has a protective role against OGD/R cell injury mainly by affecting lipids and lipid-like molecules, organic acids and derivatives, benzenoids, and other undefined metabolic pathways. HBA was associated with a total of 17 differential metabolites (Table 1). Of the differential metabolites, choline exhibited the most significant difference between the groups. Choline is a micronutrient that is mainly involved in the structural integrity of cell membranes, signal transduction, cholinergic nerve transmission, and other physiological functions [24]. Choline has an essential role in brain development, preventing neurological and metabolic damage and improving neurological and cognitive function [25]. It has been indicated that choline supplementation improves growth retardation, neurological impairment, memory impairment, and cerebral ischemia [26]. Jin et al. [27] found that oral choline administration significantly reduced cerebral ischemic injury in rats with MCAO, lowering neurological dysfunction, cerebral infarction volume, and nerve cell necrosis. Borges et al. [28] found that oral choline supplementation for 7 days significantly increased the survival rate of hippocampal neurons in the CA1 region after transient global cerebral ischemia in rats. D-glutamine has significant biological activity and is essential for oxidative stress, nitrogen metabolism, and mitochondrial function, showing great potential in nutrition and disease therapy [29,30]. Guanabenz is related to prolonging the life span of mice, delaying the onset of disease symptoms, improving motor performance and reducing the loss of motor neurons. It may also regulate the protein level of anti-apoptotic Bcl-2 and downregulate the levels of the pro-apoptotic C/EBP homologous protein, Bax and cytochrome c [31]. Luffariellolide exerts its anti-inflammatory effect by inhibiting phospholipase A2 and reducing the levels of platelet-activating factor [32]. Dimethyl sulfoxide affects impaired blood flow by inhibiting the cytotoxicity caused by excessive glutamate release. Blocking tissue factors to reduce thrombosis may also reduce intracranial pressure, tissue edema and inflammation, and scavenge free radicals [33,34]. In ischemic injury, dimethyl sulfoxide may participate in the dynamic process of promoting vascular biochemical and morphological changes by regulating the platelet system [35]. In addition, it may regulate the hyperresponsiveness of astrocytes and neuron-astrocyte structure, and thus has potentially neuroprotective properties in treating acute thromboembolic cerebral ischemia [36].

Among the metabolites induced by HBA, the most significant differential metabolite was choline, which is essential in treating cerebral ischemia. The six-member PLD enzyme superfamily (PLD1–6) is the primary means regulating choline production in vivo [37]. These enzymes exhibit a series of substrate specificities, are regulated by different molecules, and are involved in numerous cellular processes, including receptor signaling, cytoskeletal regulation, and membrane transport [38]. PLD2 is the most widely studied subtype of PLD [39]. It has been indicated that PLD2 promotes the production of choline and participates in the inhibition of apoptosis, PC12 cell differentiation, actin-based membrane dynamics, and nerve cell adhesion molecule-dependent axonal growth [38]. The upregulation of PLD2 during ischemia and hypoxia may be involved in the neuroprotective mechanism of ischemic tolerance in the hippocampi of rats, as well as the inhibition of human neuroblastoma cell death induced by chemical hypoxia [40]. Another study found that 3 h after cerebral ischemia, the number of PLD2 mRNA in the brainstem and cerebellum decreased significantly and the inhibition of PLD signal transduction was involved in the induction of apoptosis and necrosis in the cerebellum and brainstem [41]. These results suggest that PLD has an important role in neuroprotection. Consistent with previous studies, the present results indicated that the protein level of PLD2 decreased after OGD/R. However, the level of the PLD2 protein increased after HBA treatment; therefore, HBA may improve OGD/R injury by increasing the level of choline via the promotion of the expression of PLD2 protein.

Mitochondrial dysfunction is one of the markers of ischemic stroke. During cerebral ischemia–reperfusion injury, the structure and function of mitochondria are damaged, leading to pathological processes, such as oxidative stress, a disorder in energy metabolism and apoptosis [42]. The decrease in MMP is a sign of mitochondrial damage, subsequently leading to the disturbance of ATP production. Accumulation and excessive levels of ROS damage mtDNA, resulting in the loss of mitochondrial function [43]. Of note, it has been indicated that PLD2 selectively interacts with SIRT1 to increase the deacetylase activity of SIRT1 [44]. According to a previous study, the activation of the SIRT1/PGC-1α pathway is beneficial to the protection of mitochondrial function, improving cognitive impairment and inhibiting neuroinflammation in rats with chronic cerebral hypoperfusion [45]. Another study indicated that the activation of the SIRT1/PGC-1α/TFAM signaling pathway improved the mitochondrial function of dorsal root ganglion neurons to prevent diabetic peripheral neuropathy [46]. These results suggest that the SIRT1/PGC-1α pathway has a vital role in protecting the function of mitochondria. However, the possibility that HBA improves cerebral ischemic injury by protecting mitochondrial function through the PLD2/SIRT1/PGC-1α pathway has not been previously studied, to the best of our knowledge.

After OGD/R, the mitochondrial MMP and ATP levels decreased, while the number of ROS increased. After HBA treatment, the changes in these indexes improved. Therefore, it is suggested that HBA may improve the mitochondrial dysfunction induced by OGD/R. Consistent with previous studies, the results of the present study suggested that HBA improved the mitochondrial structural damage and swelling caused by OGD/R [47]. In addition, RT-qPCR indicated that HBA increased the level of mtDNA and the mRNA levels of genes related to the SIRT1/PGC-1α pathway (SIRT1, PGC-1α, NRF1, NRF2, and TFAM) in the HT22 OGD/R cell model. Similarly to the RT-qPCR results, Western blot analysis indicated that HBA promoted the expression of SIRT1/PGC-1α pathway-related proteins (SIRT1, PGC-1α, NRF1, NRF2, and TFAM). This suggested that the protective effect of HBA against OGD/R damage is related to the activation of the SIRT1/PGC-1α pathway, leading to the improvement of mitochondrial dysfunction.

In view of the importance of the significantly different metabolite choline in cognitive function, in vivo experimental research was also conducted in this study. The VD rat model was replicated by 2VO, and the improvement of cognitive function and pathological changes in hippocampal tissue in rats were observed after HBA intervention. Through the results of the Morris water maze experiment, it was found that the cognitive impairment of VD rats was improved after HBA treatment. The escape latency in the positioning navigation experiment was gradually shortened and approached the level of the sham group, and the swimming path also became relatively regular and efficient. The number of platform crossings was significantly increased, and the proportion of time spent in the target quadrant was also significantly increased [48]. The trend of the Morris water maze experiment is consistent with other references, which fully validates the recovery effect of HBA on cognitive memory [49].

Hippocampal tissue is a key structure in the brain that is closely related to learning, memory, and cognitive functions. Its normal operation plays an indispensable role in the exertion of important brain functions [50]. From the perspective of neurobiology, as a key structure in the brain, the hippocampus is deeply involved in multiple key links such as information encoding, storage, and retrieval [51]. In the learning process, through its complex neural network, the hippocampus integrates and processes new external stimuli, and converts the perceived information into storable neural representations, laying the foundation for subsequent memory consolidation [52]. For memory, the hippocampus plays a central role in the transformation from short-term memory to long-term memory. It can promote the strengthening and stabilization of connections between neurons, so that important information can be preserved for a long time [53]. In terms of cognitive functions, the hippocampus is interconnected with multiple brain regions and jointly participates in advanced cognitive processes such as spatial cognition, context recognition, and logical reasoning [54]. Studies have shown that damage to the hippocampus will significantly affect an individual’s learning ability, memory performance, and cognitive flexibility, and hinder adaptation to new environments and problem-solving. Once the hippocampal tissue is damaged, it often leads to serious disorders in learning, memory, and cognition [55]. At the same time, this study analyzes the improvement effect of HBA on the pathological changes in hippocampal tissue in VD rats by observing HE staining, the number of Nissl bodies, and TUNEL fluorescence staining. The results show that HBA can effectively alleviate the pathological changes in the hippocampal region. The arrangement of neurons tends to be neat, the morphology is improved, the neuropil structure is clear, and the infiltration of inflammatory cells is reduced. The phenomenon of a decreased number of Nissl bodies and an increased number of TUNEL-positive cells has also been significantly improved. These results reflect the positive role of HBA in ameliorating severe neuronal damage in the hippocampus of 2VO, which is consistent with the previous literature [56]. The destruction of the normal structure and function of the hippocampus is further prevented to avoid negative impacts on learning, memory, and cognitive functions. This will improve the neural microenvironment in the hippocampal region and the survival and function of surrounding normal neurons.

This study also confirmed in vivo experiments that HBA can effectively activate the protein expression in the PLD2/SIRT1/PGC-1α pathway and play a crucial role in the restoration of mitochondrial generation and function. However, we also note that excessive mitochondrial autophagy has profound and multifaceted impacts on the balance of mitochondrial generation [57]. Although autophagy can clear damaged mitochondria to a certain extent, excessive autophagy will break the quality homeostasis of the mitochondrial population and mistakenly degrade some mitochondria with potential repair possibilities, thereby hindering the generation process of new mitochondria. This will seriously disrupt the dynamic balance between mitochondrial generation and degradation [58]. At the same time, excessive autophagy causes a large number of mitochondria to be degraded, which will seriously interfere with the neuronal cell’s energy supply mechanism and hinder the generation of energy required for normal physiological activities. It will also trigger a strong stress response in the neuronal cell and greatly increase the risk of apoptosis [59]. This is very detrimental to the development of VD.

Beclin-1, LC3, and P62 play important roles in starting, marking, and connecting substrates in autophagy, respectively. They are interconnected and interact with each other, jointly constituting a complex network of autophagy regulation [60]. As an initiator of autophagy, Beclin-1 can interact with multiple proteins and promote the formation of autophagosome precursor structures, thereby initiating the autophagy process [61]. As a marker protein present on the autophagosome membrane, LC3 can reflect the number of autophagosomes and autophagy activity. At the same time, it also participates in the extension and closure of autophagosomes to ensure the normal formation of autophagosomes [62]. P62 interacts with LC3 to connect the substrate to be degraded to the autophagosome and so that they may be degraded together [63]. We analyzed the results based on previous reference data [64]. The results of this study show that after successful modeling, the increased expression of Beclin-1 and LC3 and the decreased expression of P62 indicate that autophagy has been activated. After the administration of HBA, the decreased expression of Beclin-1 and LC3 and the increased expression of P62 indicate that autophagy has been alleviated. As an important organelle for cell survival, mitochondria’s sufficient quantity and stable function play a crucial role in normal physiological activities, survival, and adaptation to various environmental changes in neuronal cells. Therefore, it is very likely that HBA promotes mitochondrial generation by activating the SIRT1/PGC-1α mitochondrial survival signal axis. At the same time, HBA also alleviates the excessive activation of autophagy after successful modeling and achieves a dynamic balance between mitochondrial generation and degradation. Thus, it maintains a stable quantity and function of mitochondria to combat the neuronal cell damage state brought about by VD.

## 4. Materials and Methods

### 4.1. Cell Culture and Drug Administration

HT22 cells purchased from Shanghai Enzyme Biotechnology Co., Ltd. (Shanghai, China), were cultured in high-glucose Dulbecco’s modified Eagle’s medium (DMEM; cat. no. C3113-0500; Biological Industries; Kibbutz Beit Haemek, Israel) containing 10% fetal bovine serum (cat. no. 04-001-1A; Biological Industries) and 1% penicillin-streptomycin solution (cat. no. C3421-0100) at 37 °C with 5% CO_2_. HBA (cat. no. AB0478-0020; Chengdu Aifa Biotechnology Co., Ltd. Chengdu, China) was dissolved in dimethyl sulfoxide diluted by DMEM at <0.1%. Cell cultures were incubated with different concentrations of HBA (0, 12.5, 25.0, 50.0 or 100.0 μM) at 37 °C for 24 h before or without OGD/R injury. The control group was cultured under the same conditions, but was not treated with drugs and OGD/R.

### 4.2. In Vitro OGD/R Model

In brief, HT22 cells were inoculated in 6-well plates with a cell density of 6.4 × 10^4^/mL and 2 mL per well. The cells were cultured in serum-free DMEM with 0 mg/L glucose, containing 10 mM of Na_2_S_2_O_4_ [65] (cat. no. R050484; Shanghai Yien Chemical Technology Co., Ltd., Shanghai, China) at 37 °C for 2 h. Na_2_S_2_O_4_ is a commonly used chemical hypoxia analog that is capable of consuming oxygen in the medium, thereby quickly and efficiently creating an anoxic environment. Subsequently, the medium was replaced with complete culture medium and cells were reoxygenated at 37 °C for 2 h. The morphology of HT22 cells was observed and images were captured under an inverted microscope (DMi1; Leica Microsystems GmbH, Wetzlar, Germany).

### 4.3. Detection of Cell Survival Rate by MTT Assay

As described previously [66], MTT (cat. no. IM0280; Beijing Solarbio Science & Technology Co., Ltd. Beijing, China) was used to determine the cell survival rate (n = 6). After 2 h of reoxygenation, 20 μL of MTT (5 mg/mL solution) was added to each well, followed by incubation at 37 °C for 4 h. The solution in each well was then replaced with 150 μL of dimethyl sulfoxide and plates were shaken for 5 min until the crystals were fully dissolved whilst avoiding exposure to light. The absorbance (A) at 490 nm was determined with a multi-function enzyme-labeling instrument (Varioskan Flash; Thermo Fisher Scientific, Inc., Waltham, MA, USA). Cell viability was calculated using the following formula: Cell viability rate = [(A_experimental group_ − A_blank_)/(A_control group_ − A_blank_)] × 100%.

### 4.4. Metabonomics Detection

HT22 cells were inoculated into a 6-well plate (6.5 × 10^4^ cells/well). The cells were divided into the OGD/R group and HBA group (OGD/R + HBA), with one sample containing two wells of cells, and six samples were collected for analysis for each group. Cells were extracted from the samples as follows: Samples were washed with pre-cooled PBS three times, underwent the removal of PBS, replacement with 800 μL of pre-cooled methanol/acetonitrile/water (2:2:1, *v*/*v*) and swirling to mix; subsequently, cells were subjected to 40-Khz ultrasound at 4 °C for 30 min, followed by incubation at 20 °C for 10 min, centrifugation at 14,000× *g* for 20 min at 4 °C, the collection of the supernatant, and vacuum drying. For MS analysis, the sample was redissolved in 100 μL of acetonitrile/water 1:1, *v*/*v* and swirled to mix. Following centrifugation at 14,000× *g* at 4 °C for 15 min, the supernatant was used for analysis.

Samples were separated on an Agilent 1290 Infinity LC UHPLC system (Agilent Technologies, Inc., Santa Clara, CA, USA) as follows: A 2.1 mm × 100 mm, 1.7-µm ACQUITY UPLC BEH Amide hydrophilic interaction chromatography column (Waters Corporation; Milford, MA, USA) was used with a column temperature of 25 °C and a flow rate of 0.5 mL/min. A sample injection of 2 μL was performed into mobile phase composition A (water + 25 mM ammonium acetate + 25 mM ammonia in water) and B (acetonitrile): the gradient elution procedure started with 95% B (0–0.5 min), was first reduced to 65% B (7.0 min), then reduced to 40% B (8.0 min), maintained at 40% B (9.0 min), and changed from 40 to 95% B (9.1 min), with a final hold at 95% B (12.0 min). Samples were placed in an automatic sampler at 4 °C throughout the analysis. To mitigate the impact of instrument detection signal, both analysis and quality control (QC) samples were analyzed in a randomized sequence without interruption. With the mixed solution serving as the QC sample, one QC sample was inserted every four analysis samples during instrumental analysis, to assess system stability and experimental data reliability.

A Triple Time Of Flight (TOF) 6600 mass spectrometer (SCIEX) was used to collect the primary and secondary mass spectra of the samples. Samples were separated using the Agilent 1290 Infinity system (Agilent Technologies, Inc.), analyzed using the Triple TOF 6600, and detected by electrospray ionization (ESI) in positive ion and negative ion modes. The setting parameters of the ESI source were as follows: atomization gas auxiliary heating gas 1 (Gas1), 60; auxiliary heating gas 2 (Gas2), 60; air curtain gas, 30 psi; ion source temperature, 600 °C; spray voltage, ±5500 V (positive and negative modes). The detection ranges of the first- and second-order mass-charge ratios were 60–1000 and 25–1000 Da, respectively, and the scanning accumulation times of the first- and second-order mass spectra were 0.20 and 0.05 s/spectrum, respectively. The second-order mass spectra were obtained in the information-dependent acquisition mode (IDA) using the peak intensity screening mode. The declustering potential voltage was ±60 V (positive and negative modes). The collision energy was 35 ± 15 eV. IDA was set with a dynamic exclusion range of isotope ions of 4 Da, with 10 fragments collected per scan.

The R package rope (www.r-project.org, accessed on 3 June 2022; version 4.2.1) was used for multivariate data analyses, which included partial least squares discriminant analysis (PLS-DA) and orthogonal PLS-DA (OPLS-DA). A 7-fold cross-validation and response replacement test was used to evaluate the robustness of the model. The variable importance in projection (VIP) was obtained from the OPLS-DA model, which was used to measure the influence and explanatory ability of the expression patterns of metabolites regarding the classification of each group of samples, and to mine the differential metabolites with biological significance. Unpaired Student’s *t*-test was used to determine the significant difference between two groups of independent samples. Metabolites with substantial differences were screened using VIP > 1 and *p* < 0.05 as the cut-off. Pearson correlation analysis was used to determine the correlation between two variables. Univariate statistics were used to analyze the differences between all metabolites (including unidentified metabolites). The differential metabolites with fold-change (FC) > 1.5 or FC < 0.67 and *p* < 0.05 were shown as volcano plots. To more comprehensively and intuitively display the relationships between samples and the differences in the expression patterns of metabolites in different samples, the R package “heatmap” was used to present the cluster analysis results of samples and metabolites in the form of heat maps. A standard in-house database (Metware Database) was searched [67]. The metabolites in biological samples were identified by analyzing the retention time, molecular weight (molecular weight error < 10 ppm), secondary fragmentation spectrum, collision energy and other information in the local database. The identification results were strictly checked and manually confirmed. Differential metabolites were screened using the OPLS-DA criteria of VIP > 1 and a *p* < 0.05. The metabolic pathways of differentially expressed metabolites were analyzed using the Kyoto Encyclopedia of Genes and Genomes (KEGG) pathway database (http://www.genome.jp/kegg/pathway.html, accessed on 24 July 2022).

### 4.5. Transmission Electron Microscopy (TEM)

After 2 h of reoxygenation, HT22 cells were collected for TEM to observe the ultrastructural changes in mitochondria (n = 3) under TEM. Cells were fixed with 2.5% glutaraldehyde at 4 °C overnight, rinsed three times with PBS (0.01 M, pH 7.2) at 4 °C (15 min each), and further fixed in 1% osmic anhydride at 4 °C for 1 h. The samples underwent gradient dehydration using propanol at concentrations of 30, 50, 70, 80, and 90% for a duration of 30 min each, followed by elution with pure acetone three times, with each cycle lasting for 30 min. After dehydration, the slices were polymerized in an incubator at 45 °C for 12 h, followed by an additional 36 h reaction at 60 °C, and ultra-thin sections of 70 nm were prepared. The ultrathin sections were stained with lead citrate for 10 min and uranyl acetate at room temperature for 30 min. The structure of mitochondria in the cells was observed and images were captured by TEM (JEM-1400Flash; JEOL, Ltd., Tokyo, Japan).

### 4.6. Detection of Mitochondrial Membrane Potential (MMP), ATP, and Reactive Oxygen Species (ROS) Levels

After 2 h of reoxygenation, the levels of MMP, ATP, and ROS were detected using kits in accordance with the manufacturers’ instructions. Using the MitoProbe™ JC-1 assay kit (cat. no. M34152; Thermo Fisher Scientific, Inc.), MMP was detected by flow cytometry. In brief, 1 × 10^6^ cells from each group were digested with 0.25% trypsin-EDTA, suspended in 1 ml of complete culture medium and incubated with 1 μL of 50 mM of carbonyl cyanide 3-chlorophenylhydrazone for 5 min at 37 °C. A total of 10 μL of 200 μM of JC-1 working solution was added to each group of cells and incubated at 37 °C for 20 min, and 2 ml of PBS was added to each tube. The tubes were centrifuged at 4 °C at 600× *g* for 3 min, the supernatant was removed, and the cell pellet was resuspended in 500 μL of PBS for detection with a flow cytometer (Beckman DxFlex; Beckman Coulter, Inc., Brea, CA, USA). A higher monomer proportion was associated with a lower MMP. An ATP content assay kit (cat. no. A095-2-1; Nanjing Jiancheng Bioengineering Institute, Nanjing, China) was used to determine the level of ATP via chemiluminescence. A ROS detection kit (cat. no. S0033S; Beyotime Institute of Biotechnology, Beijing, China) was used and fluorescence was measured at an excitation wavelength of 488 nm and an emission wavelength of 525 nm; a higher fluorescence value was associated with a higher ROS level. The levels of ATP and ROS were detected by full-wavelength scanning with a multi-function enzyme labeling instrument.

### 4.7. RT-qPCR

Following the instructions of the RNAsimple Total RNA Kit (cat. no. DP419; Tiangen Biotech Co., Ltd., Beijing, China), total RNA was extracted from HT22 cells. The GoScript RT System (cat. no. A5001; Promega Corporation, Madison, WI, USA) provided templates for fluorescence qPCR according to the manufacturer’s instructions, which was performed on a Light Cycler 96 (Roche Diagnostics, Basel, Switzerland) using Go Taq^®^ qPCR Master Mix (cat. no. A6001; Promega Corporation). The following primers were used: Sirtuin1 forward (F), 5′-GCAGGTTGCAGGAATCCAAA-3′ and reverse (R), 5′-GGCAAGATGCTGTTGCAAAG-3′; peroxisome proliferator-activated receptor γ coactivator 1α (PGC-1α) F, 5′-GCAGTCGCAACATGCTCAAG-3′ and R, 5′-GGGAACCCTTGGGGTCATTT-3′; nuclear respiratory factor 1 (NRF1) F, 5′-AGAAACGGAAACGGCCTCAT-3′ and R, 5′-CATCCAACGTGGCTCTGAG-3′; NRF2 F, 5′-ATGGAGCAAGTTTGGCAGGA-3′ and R, 5′-GCTGGGAACAGCGGTAGTAT-3′; transcription factor A, mitochondrial (TFAM) F, 5′-TCCACAGAACAGCTACCCAA-3′ and R, 5′-CCACAGGGCTGCAATTTTCC-3′; mitochondrial DNA (mtDNA) F, 5′-ACTGAATCCTAGTAGCCAACC-3′ and R, 5′-GATGGAGGCTAGTTGGCCA-3′; and β-actin F, 5′-TGCTATGTTGCCCTAGACTTCG-3′ and R, 5′-GTAACAGTCCGCCTAGAAGCAC-3′ [68,69,70,71]. Relative gene expression levels were calculated using the 2^−ΔΔCq^ method with the β-actin gene used as the internal control [72].

### 4.8. Experimental Animals

24 SPF male Sprague Dawley rats, 7 weeks old, with a body weight of 250–280 g, were ordered from Hunan Slack Jingda Laboratory Animal Co., Ltd. (Changsha, China), license number SCXK (Xiang) 2019-0004. They were kept in the SPF animal room of the Laboratory Animal Center of Yunnan University of Traditional Chinese Medicine under constant temperature and humidity. During the experiment, the animals were free to use food and water at will. All procedures involving animals were approved by the Animal Ethics Professional Committee of Yunnan University of Chinese Medicine (Ethics Review Number: R-062021088).

### 4.9. Grouping Dosing and Replication of the Model

The 24 rats were randomly divided into a Sham group, a 2VO group, an HBA-H group, and an HBA-L group. The 6 mice in each group all performed the water maze experiment. Three brain tissues from each group of six mice were used for HE staining, Nissl staining, TUNEL staining and immunofluorescence staining. The other three brains were used in Western blot experiments. According to the previous study of the research group, the effective doses of HBA were determined to be 20 mg/kg and 10 mg/kg. After modeling, the HBA-H group and the HBA-L group were administered by gavage for 30 days, once a day. The Sham group and the 2VO group were administered with normal saline in equal volumes.

All rats were randomly grouped after 1 week of acclimatization. Rats were subjected to basic anesthesia using 2.5% isoflurane followed by maintenance anesthesia with 1.5% isoflurane. The 2VO group and the treatment group underwent the separation and exposure of bilateral common carotid arteries, and their distal and proximal cardiac ends were ligated and sutured layer by layer. In the Sham group, only bilateral common carotid arteries were isolated and no ligation was performed.

### 4.10. Water Maze Experiment

The learning and memory function of rats in each group was evaluated by the water maze experiment. The swimming pool had a diameter of 150 cm, a depth of 30 cm, and a water temperature of 23–25 °C. After the end of dosing, the rats were transported from the 1st to the 4th quadrant of the water maze, and the positioning navigation training was carried out regularly every day for 5 days. The longest test time was 90 s, and the time of escape latency was recorded as 90 s for rats that exceeded the maximum test time, and 15 s were learned and memorized on the platform. The escape latency for each rat is the average of the first 5 days of the test time. On the 6th day, the space exploration experiment was carried out, the platform was removed, the rats were uniformly put into the water from the first quadrant, the trajectory recording system was turned on, and each rat was recorded for 120 s. The swimming trajectory of each rat, the number of times it crossed the platform, and the stay time in the target quadrant were recorded.

### 4.11. HE Staining

Brain tissue was fixed in 4% paraformaldehyde overnight at room temperature, paraffin-embedded, and sectioned into 5 μm slices. It was placed in an electric constant temperature drying oven and baked at 60 °C for 3 h, the conventional xylene was dewaxed, and the descending gradient ethanol was dehydrated. Hematoxylin was used for staining for 2 min. Differentiation with 1% hydrochloric acid alcohol was performed for a few seconds and then rinsed for 5 min. Eosin staining was performed for 1 min and then rinsed until residual dye is removed. The sections were mounted with ethanol gradient increment, xylene transparent for 5 min, and neutral gum. We observed the pathological changes in hippocampal tissue with an inverted phase contrast microscope at a magnification of 400×. The CA region of the hippocampus was observed. Each group made 3 slides, and from each slide, 3 fields of view were randomly selected for image capture.

### 4.12. Nissl Staining

Brain paraffin sections were routinely deparaffinized, then dehydrated with descending gradient ethanol and impregnated with Nissl staining solution for 1 h in a 56 °C incubator. The sections were washed with deionized water, placed in Nissl differentiation solution for differentiation, anhydrous ethanol dehydrated, xylene transparent, neutral gel mounted, and kept warm at 50~60 °C for 40 min. The CA region of the hippocampus was observed. Each group made 3 slides, and from each slide, 3 fields of view were randomly selected for image capture. The pathological changes in ischemic neurons were observed under an inverted phase contrast microscope under a 400× microscope, and the photometric values in three non-overlapping fields were calculated with ImageJ software.

### 4.13. Tunnel Staining

Paraffin sections were routinely dewaxed to water. We added 20 μg/mL of proteinase K without DNase at 37 °C and let it act for 25 min. After washing three times with PBS, 50 μL of TUNEL staining solution was added and incubated in the dark for 60 min. Samples were then wash three times with PBS. After mounting, observation under a fluorescence microscope was performed. A field of view was randomly selected and a picture was taken under a 400× field of view. The CA region of the hippocampus was observed. Each group made 3 slides, and form each slide, 3 fields of view were randomly selected for image capture. The rate of TUNEL-positive cells (%) = TUNEL-positive cells/Total cells × 100.

### 4.14. Immunofluorescence Staining

Paraffin sections were washed three times with PBS. A circle was drawn with an immunohistochemical pen. An amount of 0.4% Triton X-100 was added and incubates for 10 min. After washing three times with PBS, the antigen was repaired. After washing three times with PBS, 5% goat serum was added and blocked at 37 °C for 10 min. The following primary antibodies were added: LC3 (cat. no. sc-515744; 1:200; Santa Cruz Biotechnology, Inc., Santa Cruz, CA, USA), p62 (cat. no. sc-515744; 1:250; Santa Cruz Biotechnology, Inc.), and Beclin1 (cat. no. sc-515744; 1:200; Santa Cruz Biotechnology, Inc.). It was able to react overnight at 4 °C. After washing three times with PBS, the following fluorescently labeled secondary antibodies were added: Goat anti-rabbit IgG H&L (HRP) (cat. no. ab6721; 1:10,000; Abcam, Cambridge, UK) and rabbit anti-mouse IgG H&L (HRP) (cat. no. ab6728; 1:10,000; Abcam), and incubated at 37 °C in the dark for 2 h. After washing three times with PBS, 90% glycerol was added for mounting. Pictures were taken under a 400× field of view using a confocal laser scanning microscope.

### 4.15. Western Blot Analysis

Cells and brain tissues were treated with RIPA lysis buffer (PMSF/RIPA lysis buffer, 1:100) (cat. nos. ST506 and P0013C; Beyotime Institute of Biotechnology) and lysed on ice for 20 min. The protein extract was centrifuged at 12,000× *g* at 4 °C for 5 min and total protein was quantified using the bicinchoninic acid method. Samples of exactly 80 μg of protein were separated by 8% SDS-PAGE and transferred to a PVDF membrane. The membranes were incubated with the following primary antibodies at 4 °C overnight: Phospholipase D (PLD)2 (cat. no. sc-515744; 1:1000; Santa Cruz Biotechnology, Inc.), SIRT1 (cat. no. ab110304; 1:1000; Abcam), PGC-1α (cat. no. ab54481; 1:1000; Abcam), NRF1 (cat. no. 46743; 1:1000; Cell Signaling Technology, Inc., Beverly, MA, USA), NRF2 (cat. no. 16396-1-AP; 1:5000; Proteintech Group, Inc., Rosemont, IL, USA), TFAM (cat. no. sc-166965; 1:1000; Santa Cruz Biotechnology, Inc.), and β-actin (cat. no. ab8226; Abcam). The membranes were then incubated with the following secondary antibodies for 1 h at room temperature: Goat anti-rabbit IgG H&L (HRP) (cat. no. ab6721; 1:10,000; Abcam) and rabbit anti-mouse IgG H&L (HRP) (cat. no. ab6728; 1:10,000; Abcam). Protein bands were detected using enhanced chemiluminescence (ECL; cat. no. WP20005; Thermo Fisher Scientific, Inc.) and then analyzed using an automatic gel imaging system (Tanon Science & Technology Co., Ltd., Shanghai, China) and ImageJ Pro Plus 6.0 software (Media Cybernetics Image Technology Inc., Rockville, MD, USA).

### 4.16. Statistical Analysis

GraphPad Prism 9.0.0 software (GraphPad Software; Dotmatics, Boston, MA, USA) was used for statistical analysis. The data followed a normal distribution. If the variance was homogeneous, one-way ANOVA followed by the Bonferroni multiple-comparisons test was used. If the variance was non-homogeneous, Dunnett’s T3 multiple-comparisons test with Welch’s ANOVA was used. Data are expressed as the mean ± standard error of the mean. *p* < 0.05 was considered to indicate a statistically significant difference [73].

## 5. Conclusions

The purpose of this study was to explore the metabolic characteristics and potential mechanisms of HBA in the treatment of cerebral ischemia, and to seek new drug targets and strategies for the treatment of cerebral ischemia. HBA, as a small molecule of fat-soluble substance, readily crosses biological barriers. This will be easily absorbed and distributed in the body to the site of injury. Through the construction of cell and animal models, the use of metabolomics technology, and a variety of detection methods, it was found that HBA can effectively improve the cognitive impairment after cerebral ischemia in rats. HBA significantly improved the cognitive memory function of VD rats; shortened the escape latency; increased the number of platform crossings and the target quadrant residence time in the Morris water maze experiment; improved the histopathological changes in the hippocampus; reduced neuronal damage, inflammatory cell infiltration, and apoptosis; regulated the protein expression of the PLD2/SIRT1/PGC-1α pathway in vivo; inhibited excessive autophagy; and maintained the balance between mitochondrial production and degradation. HBA is protective against OGD/R-damaged HT22 cells, and its concentrations from 12.5 to 100 μM are non-cytotoxic and increase cell viability and morphology. Metabolomic analysis showed that HBA mainly played a role by influencing multiple metabolic pathways and 17 differential metabolites, among which choline was the most critical, and HBA could upregulate PLD2 and promote choline production. At the same time, HBA can improve mitochondrial dysfunction, increase the expression of related genes and proteins through the PLD2/SIRT1/PGC-1α pathway, and regulate mitochondrial membrane potential, ATP, ROS, and other indicators.

However, there are limitations to this study. Considering the central role of mitochondria in a variety of neurodegenerative diseases, these findings on HBA may be of great significance for the development of new therapeutic strategies, especially in the treatment of mitochondrial dysfunction-related diseases. Based on the current research results, future studies will continue to deepen the understanding of the mechanism of action of HBA and explore its potential application in other mitochondria-related diseases.

## Figures and Tables

**Table 1:** Screening results of differential metabolites in the OGD/R and HBA groups.

Metabolite	RT, s	*p*-Value	VIP	Fold-Change	Direction of Change
Choline	386.536	0.190 × 10^−3^	1.677	1.469	Up
D-glutamine	424.808	0.917 × 10^−3^	1.702	2.319	Up
Guanabenz	396.218	0.664 × 10^−2^	1.288	1.296	Up
3,3,4-trimethyl-1-1-[(tetrahydro-2H-pyran-4-yl) methyl]-1H-indol-3-yl-4-penten-1-one	277.747	0.816 × 10^−2^	1.118	1.521	Up
Luffariellolide	34.142	0.176 × 10^−1^	1.061	1.989	Up
Dimethyl sulfoxide	57.173	0.224 × 10^−1^	2.131	2.231	Up
1-Oleoyl-sn-glycero-3- phosphocholine	182.801	0.336 × 10^−1^	5.031	3.742	Up
Monoelaidin	170.006	0.356 × 10^−1^	1.014	2.330	Up
1-(1z-hexadecenyl)-sn-glycero-3- phosphocholine	183.994	0.433 × 10^−1^	1.000	0.714	Down
Tuberostemonine	33.095	0.442 × 10^−1^	1.096	0.805	Down
Phe-gly	44.287	0.171 × 10^−2^	1.646	1.100	Up
Diclofenac	397.075	0.375 × 10^−2^	1.106	1.228	Up
9-OxoODE	26.522	0.145 × 10^−1^	4.179	1.153	Up
Tetrahydrocorticosterone	29.828	0.172 × 10^−1^	2.628	3.108	Up
11,12-dihydroxy-5z,8z,14z- Eicosatrienoic acid	55.090	0.180 × 10^−1^	1.486	1.845	Up
Sulindac	48.197	0.216 × 10^−1^	2.856	1.674	Up
Prostaglandin E2	125.712	0.380 × 10^−1^	1.430	1.437	Up
Myristic acid	50.766	0.471 × 10^−1^	3.537	1.357	Up

## Data Availability

The raw data supporting the conclusions of this article will be made available by the authors on request. The metabolomics data presented in the study are openly available in Metabolights at accession number MTBLS7756.
