# Correction: Xiao et al. Mechanism of P-Hydroxy Benzyl Alcohol Against Cerebral Ischemia Based on Metabonomics Analysis. *Int. J. Mol. Sci.* 2025, *26*, 317

**DOI:** 10.3390/ijms27010145

**Published:** 2025-12-23

**Authors:** Tian Xiao, Xingling Yu, Jie Tao, Jiaoyang Tan, Zhourong Zhao, Chao Zhang, Xiaohua Duan

**Affiliations:** Yunnan Key Laboratory of Dai and Yi Medicines, Yunnan University of Chinese Medicine, Kunming 650500, China; xiaot7107@163.com (T.X.); 18212943765@163.com (X.Y.); 19188572767@163.com (J.T.); 13350039866@163.com (J.T.); zhaozr_cpu@163.com (Z.Z.); zhangc19@21cn.com (C.Z.)

In the original publication [1], reference 71 has been retracted. The author has reviewed and decided that this reference should be removed. With this correction, the order of some references has been adjusted accordingly. The authors state that the scientific conclusions are unaffected. This correction was approved by the Academic Editor. The original publication has also been updated.
